# Root traits of dryland winter wheat (*Triticum aestivum* L.) from the 1940s to the 2010s in Shaanxi Province, China

**DOI:** 10.1038/s41598-020-62170-0

**Published:** 2020-03-24

**Authors:** Yingying Sun, Suiqi Zhang, Wei Chen

**Affiliations:** 1 Shaanxi Province Land Reclamation Engineering Technology Research Center, Shaanxi Provincial Land Engineering Construction Group Co., Ltd., Xi’an, 710075 China; 2 State Key Laboratory of Soil Erosion and Dryland Farming on the Loess Plateau, Northwest A&F University, 26# Xinong Road, Yangling, 712100 China; 3 Shaanxi Key Laboratory of Disaster Monitoring and Mechanism Simulation/School of Geography and Environment, Baoji University of Arts and Sciences, Baoji, 721013 China

**Keywords:** Plant evolution, Plant stress responses

## Abstract

Eight cultivars of winter wheat (*Triticum aestivum* L.) adapted to dryland conditions that have been historically planted in Shaanxi Province, China, were grown in plots with irrigation and drought treatments during the growing seasons of 2010–2012 to characterize the changes in root system traits and water use efficiency during the replacement of cultivars. The results showed that the overall root size of dryland wheat cultivars in Shaanxi Province changed with the planting decade. Modern cultivars developed after the 2000s had larger root surface areas than older cultivars under the drought treatment, especially at soil depths of 0–40 cm. However, the total water consumption throughout the stages showed no obvious changes among cultivars. The yield significantly increased with the planting decade, and the water use efficiency showed an average increase of 47.07% from the earliest to the most recent studied cultivar. Water stress promoted larger root sizes than those found in the irrigation treatment, especially at maturity. A trend toward a lower stress susceptibility index was observed over the decades, indicating that the sizes of modern cultivar roots increased less in the drought treatment than in the irrigation treatment. Both the roots and yields of the landrace cultivar from the 1940s showed low sensitivity to drought and better adjustment between the different water conditions. The study revealed that (1) modern wheat cultivars in Shaanxi Province possess higher water use efficiencies and decreased drought resilience and (2) the selection of ideal root traits should consider stable yields under different water conditions.

## Introduction

Since Aamodt and Johnston (1936) promoted the importance of root growth for wheat yield (*Triticum aestivum* L.)^1^, the relationship between root traits and the yield potential of wheat has been the emphasis of research by agronomists and ecologists^2,3^. Early studies suggested that larger and deeper root systems were beneficial for greater water absorption^4,5^. However, studies in recent years have indicated that appropriate root sizes and better root physiological function could promote nutrient absorption and lodging resistance in wheat^6^, which contribute to high yield production^7,8^, while a root system that is too large results in root redundancy and causes an imbalance in the distribution of dry matter.

There is continuous debate regarding the value of root size in dryland crops due to the diversities of environments, planting seasons and precipitation during growing seasons. It is generally considered that crops that mainly depend on presowing precipitation storage in the soil should reduce water use during the vegetative stage, as lower root growth rates in earlier stages are favorable for water use in the later stages of reproductive growth^9,10^. Conversely, larger root systems would be more beneficial for water absorption and utilization in areas with more precipitation^11,12^. A study by Nakhforoosh *et al*. (2014) proved that more roots near the surface soil are conducive to the full absorption and utilization of soil water^13^, while Kirkegaard *et al*.^14^ and Pask and Reynolds (2013)^15^ considered that deep soil water after anthesis would be better utilized by increases in the deep root ratio. In conclusion, the selection of the ideal root system structure should consider the metabolic costs of the production and maintenance of root tissues alongside the capacity for capturing resources^16^.

There are significant differences in the root traits among different genotypes of the same crop^13,17,18^. Spring wheat cultivars bred after 1990s possess smaller root systems than earlier and landrace cultivars^19^ as a result of the introduction of dwarf genes^20^. Studies on barley have confirmed that landrace cultivars have larger root systems and longer root lengths than modern cultivars^21,22^. Soil moisture is one of the conditions that most affects root growth. A nonirrigated condition was recorded to result in greater wheat root growth in India^23^, and wheat plants grown under moderately dry conditions developed root branching as an effective adaptation in Japan, although they were unable to recover the ability to absorb water after severe water stress^24^.

Fischer and Maurer (1978) investigated the use of a stress (drought) susceptibility index (SSI/DSI) to compare the drought resistance ability during cultivar replacement^25^, which characterizes the yield stability between different irrigation environments. There are many reports in the literature on the use of SSI or DSI for identifying genotypes with yield stability in moisture-limited environments^26–28^.

However, due to the strict requirements for manpower, material resources and planting time in field trials, current studies on the root growth differences among wheat cultivars have mostly been carried out in potted or soilless cultures. The reliability of these research results is undoubtedly affected by the lack of realistic conditions and repetition over different planting years^3,20^. Ecologists infer that agronomists and breeders might unconsciously select wheat varieties with reduced root redundancy to increase yield, especially during the breeding process for winter wheat adapted to dryland conditions^29,30^.

Based on this selection along with the replacement of dryland wheat cultivars in Shaanxi Province of China, modern cultivars should show smaller root systems, decreased root/shoot ratios and increased water use efficiencies for grain production. This study is focused on the verification of this hypothesis using irrigation and drought treatments in field plot experimental conditions.

## Materials and methods

### Plant material

Eight dryland wheat cultivars that are considered to be drought tolerant and have been widely planted in Shaanxi Province were selected (Table 1)^31^. These varieties are representative of the cultivars planted in different decades. Mazha of the 1940s is a landrace cultivar in Shaanxi Province that was once widely used as a mainstay parent. The cultivated areas of Bima 1, Fengchan 3, Taishan 1 and Xiaoyan 6 exceed 667 thousand ha. Jinmai 33, Changwu 134 and Changhan 58 are all well-known dryland wheat cultivars in Shaanxi Province and are often selected to study drought resistance. According to the historical background information, the yields were expected to increase with the planting decades of the selected cultivars, while the height was expected to decrease. The actual yield and height trends were described in a paper published in *Field Crops Research*^32^ and were shown to be basically consistent with the expected trends.

**Table 1:** Representative cultivars of dryland winter wheat in Shaanxi Province from the 1940s-2010s and their historical background information.

Cultivar	Decade	Pedigree	Dwarf gene	Breeding site	Recorded grain yield (kg ha^−1^)	Height (cm)
Mazha	1940s	Landrace	None	Shaanxi Province	3526	109
Bima 1	1950s	Mazha/Biyu	None	Shaanxi Province	4089	129
Fengchan 3	1960s	Danmai 1/Xinong 6028 × Bima 1	None	Shaanxi Province	4954	120
Taishan 1	1970s	54405(Bima 4 × Zaoshu 1)/Ourou	*Rht-D1b*	Shandong Province	4979	95
Xiaoyan 6	1980s	(ST2422 × 464)/Xiaoyan 96	*Rht-B1b* + *Rht8*	Shaanxi Province	5247	90
Jinmai 33	1990s	Pingyang 79391 ((Naixue × 5017) × 036 × 76–1256)/Pingyang 76262	None	Shanxi Province	4265	100
Changwu 134	2000s	[(Changwu 131 Triticale generation 96)F1/Changwu 131]F4/(Jinghua 3/NS2761)F1	*Rht-B1b*	Shaanxi Province	5164	85
Changhan 58	2010s	Changwu 112/PH82–2	*Rht-B1b*	Shaanxi Province	5281	78

### Experimental site condition

Field experiments were conducted in Yangling, Shaanxi Province, Northwest China (34° 16′ 56.24″ N, 108° 4′ 27.95″ E; 460 m above sea level) over 2 winter–spring growing seasons (October–June of the following year between 2010 and 2012). The experiment setup is same as that in another article published by the author^32^. The experiment area is in the temperate monsoon zone with a semihumid climate, an annual average temperature of 10.7–13.7 °C, and an annual total precipitation of 552.6–663.9 mm. The soil consists of Earth-cumuli-Orthic Anthrosols (Chinese soil taxonomy,1995) with a deep profile and is considered suitable for crop production. Mung beans were planted during the fallow period of each year, and irrigation was provided to regulate the soil water and fertilization. In the 2 m soil profile, the average field capacity was 28% (V_moisture_/V_soil_) before each planting season^32^.

### Experimental design

The cultivars were manually planted in plots (2.2 × 3.3 m per plot; 11 rows, 20 cm apart; plant spacing of 2 cm). The plots were arranged in randomized blocks with three replicates. Seeds of the experimental cultivars were planted in the field on October 7, 2010, and October 10, 2011, and harvested from the end of May to the beginning of June in 2011 and 2012, respectively. Fungicides and pesticides were applied at shooting and grain filling to prevent attacks by diseases and pests. Bamboo poles (50–200 cm long) were used to prevent lodging in cultivars with no dwarf genes so that the ultimate yield potential could be reached^32^.

**Figure 1:**
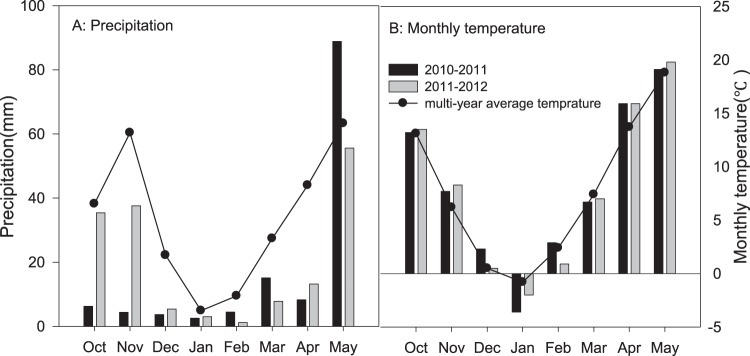
Precipitation and monthly temperature during the experimental period (October–May of the following year) between 2010 and 2012 compared with the long-term means (1956–2005) at the experimental site.

Two water treatments were implemented (Table 2): one with normal precipitation and two irrigation events (irrigation, Ir), provided at the tillering stage and at the elongation stage, and the other treatment with no precipitation following the tillering stage (drought, D). A movable shed was used to block precipitation. The precipitation during the growing seasons of the two years was recorded with a rain gauge, and the monthly temperature was obtained from local weather stations (Fig. 1)^32^.

**Table 2:** Total precipitation during the growth period of 2010–2012 (mm) and the irrigation supplement in the irrigation treatment (mm).

Treatments	Drought treatment	Irrigation treatment		
Experimental seasons	Precipitation	Precipitation	Irrigation supplement at tillering stage	Irrigation supplement at elongation stage
2010–2011	18.6	133.6	70.0	70.0
2011–2012	82.8	159.2	70.0	80.0

### Measurements

#### Determination of root traits

Drills with diameters of 9 cm were used to collect soil containing wheat root from the plots at anthesis and maturity. Based on the common root drill sampling points (Kumar *et al*. 1993), three points were selected in each plot, two points in the lines (one with a plant as the center and the other with the center between two plants as the center; the distance between the centers of the two points was kept between 8 and 16 cm) and the third in the center between lines. One soil sample was taken for every 10 cm step at a depth up to 2 m. Roots were handpicked from the soil and rinsed with sieves of mesh size 0.25 mm, and the root surface area was obtained using a winRHIZO root image analysis system and weighed after drying. The average of three points in one plot represents the specific root characteristic parameters, and the average of three plots represents the root characteristic parameters of the specific cultivar under the same treatment.

At maturity in both seasons, four central rows (1 m long) were harvested and weighed to determine the shoot weight, and the root/shoot ratio was calculated using the following equation:

$${\rm{Root}}/{\rm{shoot}}\,{\rm{ratio}}={\rm{R}}/{\rm{S}}$$

where R is root weight (kg ha^−1^) and S is shoot weight (kg ha^−1^).

#### Determination of water consumption and water use efficiency

The soil moisture conditions were recorded with a CNC503B neutron moisture gauge (China) before sowing and at anthesis and maturity stages. One sample was taken for every 10 cm step at 0–1 m depth and every 20 cm depth at 1–2 m depth. The soil moisture content of each plot was calculated using the weighted average value from the depth of 0–2 m.

Soil moisture was converted into soil water storage in mm:

$${{\rm{W}}}_{{\rm{S}}}={\rm{\theta }}\cdot {\rm{h}}/100$$

where θ is soil moisture content (%), h is soil depth (mm).

Evapotranspiration (ET) was calculated as the change in water storage in the soil profile plus precipitation between sowing and harvest. The experimental plots were flat, and negligible runoff was assumed. For the rainfall and soil combinations under study, rainfall infiltration was mostly limited to the top 2 m. The water use efficiency (WUE) was calculated as grain yield divided by ET.

### Measurements

The significance of cultivar effects was determined by SAS 8.1 using an analysis of variance (SAS, USA). Correlations between phenotypic traits and yield elements were determined using Pearson’s test. SPSS 19.0 software was used to perform the analyses.

The stress susceptibility index (SSI) of root traits was calculated using the following equation:

$$SSI=(1-{{\rm{R}}}_{{\rm{D}}}/{{\rm{R}}}_{{\rm{Ir}}})/{(1-{\bar{R}}_{D}/{\bar{R}}_{{\rm{Ir}}})}^{25}$$

where R_D_ is the root trait (root weight and root surface area) of the cultivar under drought; R_Ir_ is the root trait of the cultivar under irrigation; ${\bar{R}}_{D}$ and ${\bar{R}}_{{\rm{Ir}}}$ are the mean root traits of all cultivars under the drought and irrigation conditions, respectively.

## Results

### Root biomass distribution characteristics and root/shoot ratio

Increases in grain yield were observed when all 8 cultivars released in different years were planted in the same habitat. The yield under the drought treatment was significantly lower than that under the irrigation treatment, while no significant changes in the aboveground biomass were observed for either treatment (Table 3). Detailed discussions about the results are provided in the previous work (Sun *et al*. 2014).

**Table 3:** Yield and aboveground biomass of the cultivars during 2010–2011, 2011–2012 and 2012–2013.

Decades	Yield (kg ha^−1^)				Aboveground biomass (kg ha^−1^)			
	2010–2011		2011–2012		2010–2011		2011–2012	
	Ir	D	Ir	D	Ir	D	Ir	D
1940s	3820b	3720a	4633b	4229c	13133a	11600a	29721a	15954a
1950s	4637ab	3480a	5954ab	5362bc	16333a	11583a	20208b	18604a
1960s	4540ab	3670a	5937ab	5250bc	18092a	12150a	21779b	17483a
1970s	4703ab	3717a	4554a	6683ab	14558a	10750a	21763b	18908a
1980s	5860ab	3893a	7354a	6270ab	13133a	11192a	23900b	19358a
1990s	6010a	4133a	6295ab	7112a	16333a	12796a	20963b	20621a
2000s	5457a	4063a	7350a	6204ab	16717a	11021a	21292b	16979a
2010s	5267a	3810a	7504a	6116ab	14558a	10250a	24213b	17817a
*SE*	262	76	415	326	633	284	1080	519

Means followed by the same letter within a column were not significantly different at P = 0.05.

**Figure 2:**
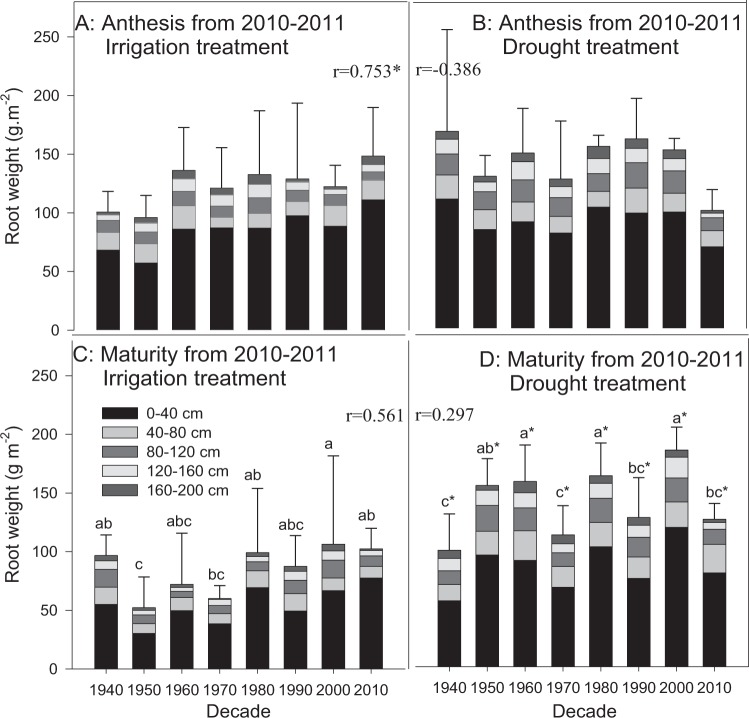
Root weight distribution at anthesis and maturity from 2010–2011. The data represent total root weight at the depth of 0–2 m with means ± SE and N = 3. Different colors on the histograms represent different soil layers. The means of cultivars followed by the same lowercase letter were not significantly different at P = 0.05 within the same water treatment; *the means of a given cultivar were significantly different at P = 0.05 between the irrigation and drought treatments. *With the *r* value indicates that the correlation between the root trait and the planting decade was significant at P = 0.05.

**Figure 3:**
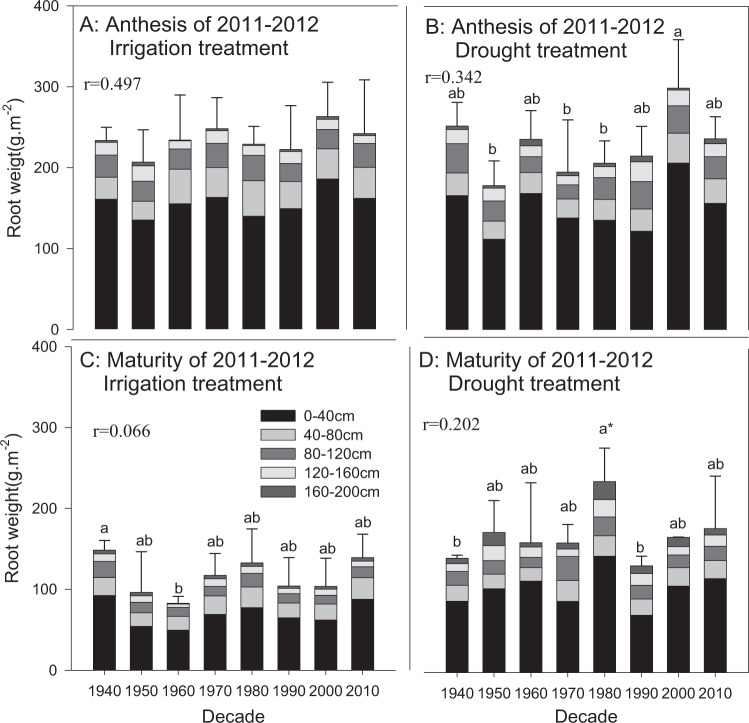
Root weight distribution at anthesis and maturity from 2011–2012. The data represent total root weight at the depth of 0–2 m with means ± SE and N = 3. Different colors on the histograms represent different soil layers. The means of cultivars followed by the same lowercase letter were not significantly different at P = 0.05 within the same water treatment; *the means of a given cultivar were significantly different at P = 0.05 between the irrigation and drought treatments. The *r* value indicates the correlation between the root trait and the planting decade.

Root weight is the most commonly used indicator to reflect root growth conditions. According to the maximum root system depth data of all cultivars of the two experimental seasons, more than 50% of the root sample points of the cultivars from the 1970s, 1980s, 2000s and 2010s did not extend to 200 cm and were typically between 150 cm and 190 cm (data not shown); the root sample points of other cultivars all reached 200 cm. No significant influence of water condition on root weight was observed at anthesis of either growing season (Figs. 2 and 3). A significant difference between the irrigation and drought treatments was observed at maturity in 2010–2011 (P < 0.05), but not in 2011–2012, except for Xiaoyan 6 from the 1980s. Compared to the irrigation treatment, the root weight at maturity showed substantial increases at depths of 0–40 cm in the drought treatment, while minor changes in the root weight ratio at different depths from 0–200 cm were observed.

The root weights of wheat cultivars released between the 1940s and 2010s showed slight increases with decade at anthesis and maturity during both growing seasons (Figs. 2 and 3), and there was a significant increase in the irrigation treatment during 2010–2011 (r = 0.753, P < 0.05). The root weights of most experimental cultivars decreased from anthesis to maturity as the fibrous root system is susceptible to wilt. In contrast, the root weights of the cultivars from the 1950s, 2000s and 2010s increased in the drought treatment, mainly at the depths of 0–40 cm and 120–160 cm. The differences between cultivars were larger at maturity than at anthesis. Changwu 134 from the 2000s exhibited the maximum root weight at maturity among all cultivars, with weights of 106.4 and 184.7 g·m^−2^ in the irrigation and drought treatments, respectively. Cultivars from the 1950s exhibited the smallest root weights in the irrigation treatment, and those from the 1940s exhibited the smallest root weights in the drought treatment, with weights of 52.19 and 99.28 g·m^−2^, respectively.

**Figure 4:**
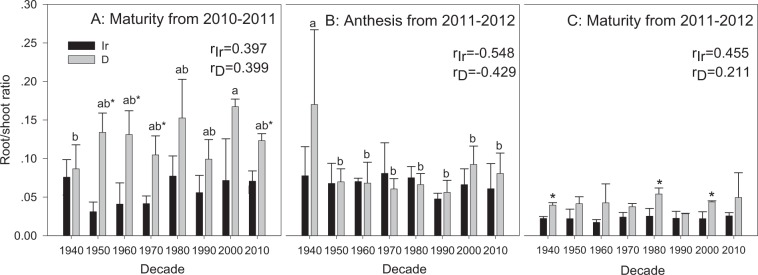
Root/shoot ratios from 2010–2011 and 2011–2012. The data represent means ± SE and N = 3. The means of cultivars followed by the same lowercase letter were not significantly different at P = 0.05 within the same water treatment. r_Ir_ and r_D_ represent the correlation between the root trait and the planting decade under the irrigation (Ir) and drought (D) treatments, respectively.

Slight increases in the root/shoot ratio at maturity during both 2010–2011 and 2011–2012 were observed, although the increases were not significant (P > 0.05, Fig. 4). Changwu 134 from the 2000s had the largest root/shoot ratio in 2010–2011 of 0.17, which was twice that of the cultivar from the 1940s (Fig. 4A). No data at anthesis from 2010–2011 were shown as the aboveground biomass value was not recorded. The root/shoot ratio decreased with decade at anthesis from 2011–2012, and the landrace cultivar from the 1940s had a ratio of 0.17 in the drought treatment, which was significantly greater than that of the other cultivars (P < 0.05, Fig. 4B). The root/shoot ratios of most cultivars were higher in the drought treatment than the irrigation treatment, especially at maturity. In addition, since the precipitation in 2010–2011 was less than that in 2011–2012 (Table 2), the aboveground biomass of all cultivars in 2010–2011 was significantly lower than that in 2011–2012 (Table 3); however, the roots may have grown larger to absorb more water. Therefore, the root weight of all cultivars in 2010–2011 was generally higher than that in 2011–2012 (Fig. 2), resulting in the root/shoot ratio of all cultivars in 2010–2011 being generally higher than that in 2011–2012 (Fig. 4).

### Root surface area distribution characteristics

**Figure 5:**
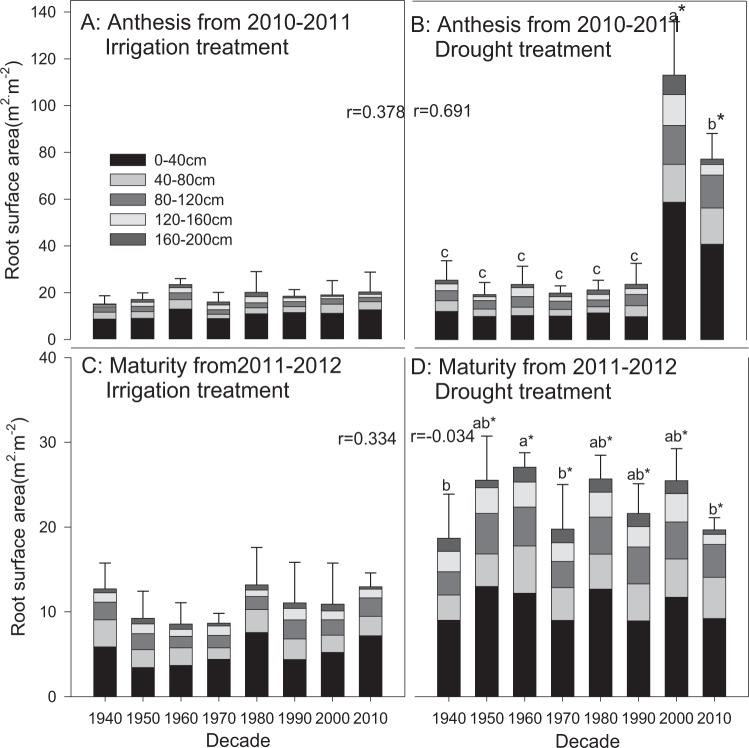
Root surface area distribution at anthesis and maturity from 2010–2011. The data represent total root surface area in the depth of 0–2 m with means ± SE and N = 3. Different colors on the histograms represent different soil layers. The means of cultivars followed by the same lowercase letter were not significantly different at P = 0.05 within the same water treatment; *the means of a given cultivar were significantly different at P = 0.05 between the irrigation and drought treatments. The *r* value indicates the correlation between the root trait and the planting decade.

**Figure 6:**
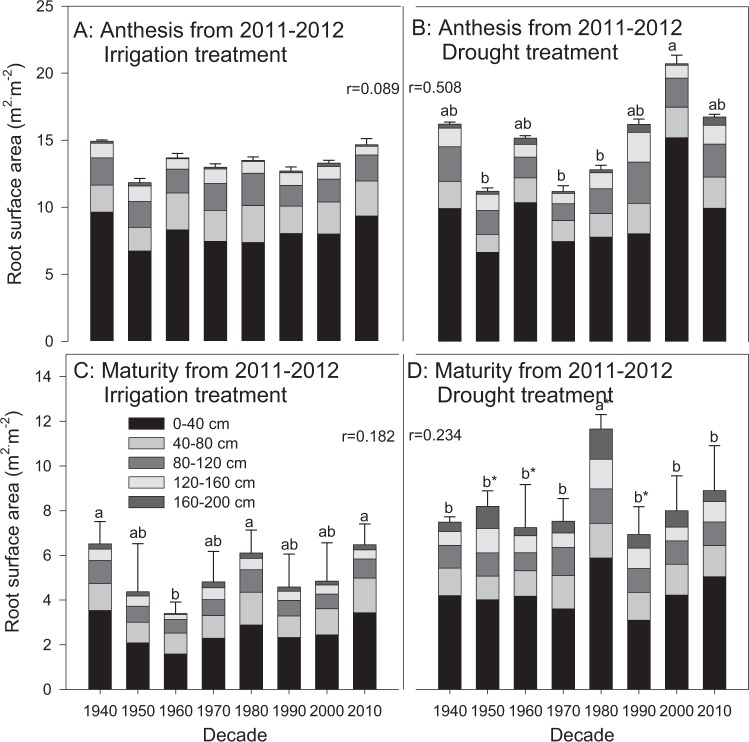
Root surface area distribution at anthesis and maturity from 2011–2012. The data represent total root surface area in the depth of 0–2 m with means ± SE and N = 3. Different colors on the histograms represent different soil layers. The means of cultivars followed by the same lowercase letter were not significantly different at P = 0.05 within the same water treatment; *the means of a given cultivar were significantly different at P = 0.05 between the irrigation and drought treatments. The *r* value indicates the correlation between the root trait and the planting decade.

No significant differences in root surface area between the irrigation and drought treatments were found in any cultivars from the 1940s to 1990s at anthesis in 2010–2011 and 2011–2012 (Figs. 5 and 6). However, the total root surface areas at the depth of 0–2 m of the cultivars from the 2000s and 2010s in the drought treatment at anthesis were 113.3 and 77.4 m^2^·m^−2^, respectively, during 2010–2011, which were significantly higher than those from the other cultivars, primarily as a result of the greater root surface areas at depths of 0–40 cm (Fig. 5B). Drought increased the root surface area of all cultivars at maturity during both growing seasons, mainly due to the increase at the depth of 0–40 cm (P < 0.05). Slight increases with decade were found in most cases (P > 0.05). The root surface area of most cultivars decreased from anthesis to maturity.

### SSI of root traits and the correlation between root traits and yield

SSI was used to measure the sensitivity of root traits to water conditions in the tested wheat cultivars. Generally, a positive value means that the response of the cultivar to water is consistent with the average response of all tested cultivars. The further the value deviates from 1, the stronger the sensitivity of this cultivar to water is. In this study, since the root characteristics of all cultivars were high under drought conditions in most cases, and the average value of all varieties was generally the same (Figs. 2–6), a greater positive deviation of the SSI value of wheat from 1 indicated a higher increase in the values for the root traits of this cultivar under drought conditions.

**Figure 7:**
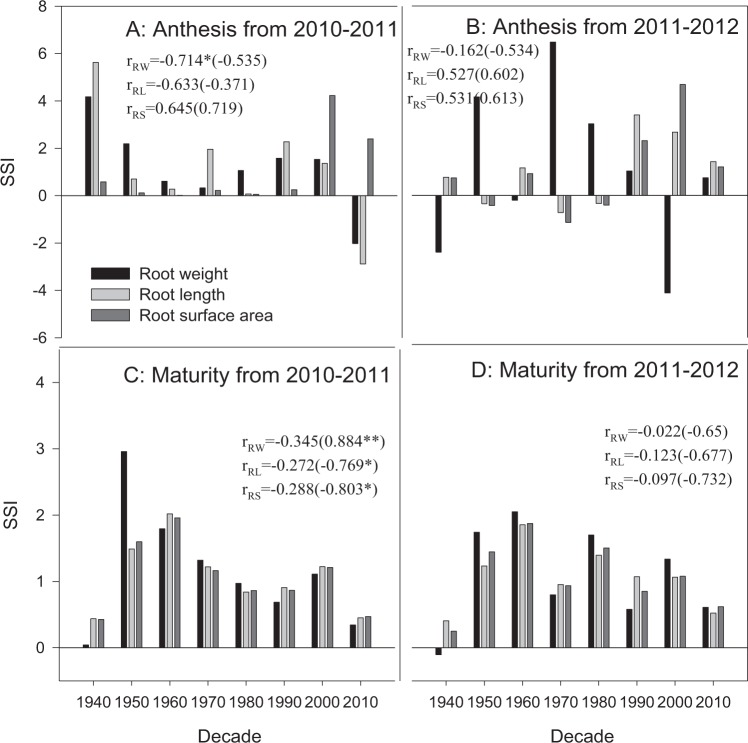
SSI of root weight and surface area during 2010–2011 and 2011–2012. Numbers in the brackets represent the correlation index between the root traits without the cultivar from the 1940s and planting decades. The data with r present the correlation between SSI and decades for all cultivars; the data in parentheses present the correlation between SSI and decades for cultivars since 1950s. *With the *r* value indicates that the correlation between the root trait and the planting decade is significant at P = 0.05; **indicates significance at P = 0.01.

Most cultivars showed positive SSI values at anthesis in both experimental seasons, and no regular changes in the SSI values of the root traits were observed with decade. The same cultivars showed different results on the different root traits in different growing seasons (Fig. 7). The root weight SSI of Changhan 58 (2010s) was −2.02 at anthesis in 2010–2011, which is less than zero, while the root surface area SSI exhibited a positive value of 2.40. The root weight SSI of Mazha (1940s) at anthesis in 2010–2011 was 4.18, which was the highest among all cultivars, but it decreased to −2.40 in 2011–2012, which was the lowest.

At maturity in both 2010–2011 and 2011–2012,the root trait SSI values of Mazha (1940s) were all less than 1 and even less than 0, which were the lowest values among all experimental cultivars (Fig. 7C,D). The SSI values of all root traits decreased significantly from the 1950s to the 2010s, especially in the growing season of 2010–2011. The SSI of Changhan 58 (2010s) was below 1, which was lower than all cultivars between the 1950s and 2000s.

For the experimental cultivars under both irrigation and drought treatments in both seasons, root weight, root surface area and root/shoot ratio showed no significant correlation with yield (Table 4).

**Table 4:** Correlation between root traits and yield of the cultivars.

r with yield	Experimental seasons	2010–2011			2011–2012		
		Root weight	Root surface area	Root/shoot ratio	Root weight	Root surface area	Root/shoot ratio
Anthesis	Ir	0.561	0.335	/	0.155	0.022	−0.48
	D	0.362	0.455	/	−0.186	−0.43	−0.465
Maturity	Ir	0.38	0.287	0.27	−0.028	0.022	0.279
	D	0.12	−0.064	0.15	0.119	0.117	−0.187

### Water use characteristics

**Figure 8:**
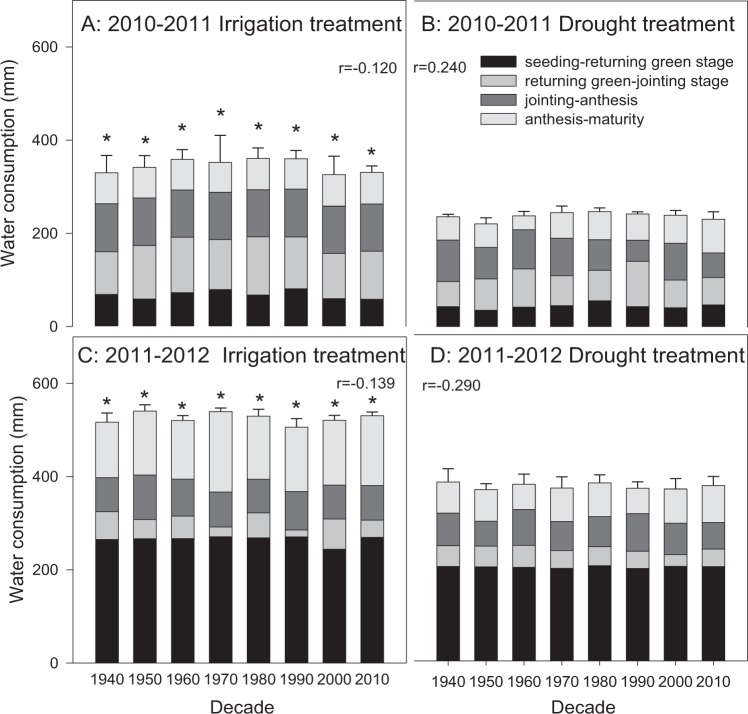
Water consumption during different growth periods during 2010–2011 and 2011–2012. The data represent total water consumption at the depth of 0–2 m with means ± SE and N = 3. Different colors on the histograms represent different soil layers. *The means of a given cultivar were significantly different at P = 0.05 between the irrigation and drought treatments.

The total water consumption amount ranged between 320 and 360 mm in the irrigation treatment in 2010–2011 (Fig. 8A) and was approximately 240 mm in the drought treatment (Fig. 8B); however, all cultivars showed no significant differences in the same treatment. There was a significant increase in the water consumption amount in 2011–2012 compared to 2010–2011, which mostly resulted from the increase during the seeding-returning tillering stage (Fig. 8C,D) when the difference between the irrigation and drought treatments increased to more than 150 mm, but a significant difference between cultivars was not found.

**Figure 9:**
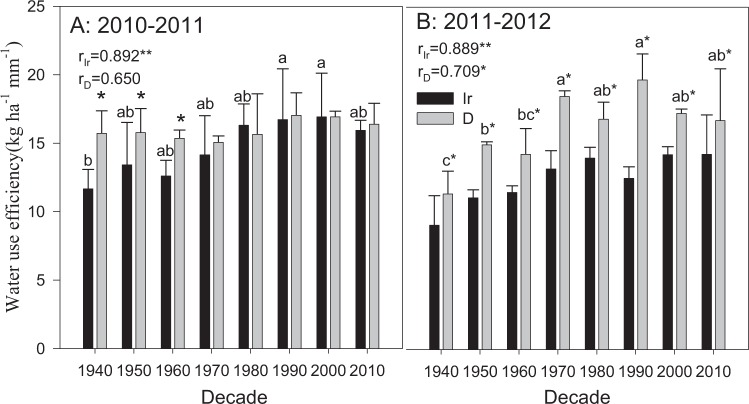
Water use efficiencies in 2010–2011 and 2011–2012. The data represent water use efficiencies with means ± SE and N = 3. The means of cultivars followed by the same lowercase letter were not significantly different at P = 0.05 within the same water treatment; *the means of a given cultivar were significantly different at P = 0.05 between the irrigation and drought treatments. r_Ir_ and r_D_ represent the correlation between the root trait and the planting decade under irrigation (Ir) and drought (D) treatment respectively, and *with the *r* value indicates that the correlation is significant at P = 0.05; **indicates significance at P = 0.01.

In 2010–2011, the WUE values of Jinmai 33 (1990s) were 16.7 and 17.0 kg·ha^−1^ in the irrigation and drought treatments, respectively, which were the highest among all cultivars (Fig. 9). There was a significant increase in the WUE with decade in the irrigation treatment (r_Ir_ = 0.892, P < 0.01); the increasing trend was less obvious in the drought treatment (r_D_ = 0.650, P > 0.05). The WUE values of only the cultivars between the 1940s and 1960s in the drought treatment were significantly higher than those in the irrigation treatment, while other cultivars were not obviously affected by the irrigation conditions. In 2011–2012, the difference in WUE increased between water treatments and significantly increased with decade (r_Ir_ = 0.889, P < 0.01; r_D_ = 0.709, P < 0.05).

## Discussions

Roots are the direct water perception and absorption organ in wheat, and root growth has been found to have a close relationship with soil water content^33^. Root growth also reflects the soil water conditions^34^, as too much or not enough soil water are both adverse conditions for root growth^35^. In the growing seasons of 2010–2011 and 2011–2012, there was consistently abundant precipitation during the bean rotation period and supplementary irrigation; thus, there was sufficient soil moisture that exceeded 30% before sowing in both growing seasons. This condition resulted in the consequence that the root traits (root weight and root surface area) showed no significant differences between the irrigation and drought treatments. At maturity, roots grew more exuberantly in the drought treatment than the irrigation treatment, especially at the surface from 0–40 cm depth, which indicated that roots might overgrow to absorb more water from the soil to overcompensate for slight water stress conditions^36^. However, the production from root overgrowth did not counteract the carbohydrates consumed, which eventually led to a significant decrease in the yield in the drought treatment compared to the irrigation treatment^32^. As a mild level of water stress was observed under the drought treatment in this research, the root weight and root surface area of all cultivars does not reflect the response in more arid environments; therefore, the results may differ from those of other studies, as the cultivars might possess less root weight and root length under severe drought stress^37^.

Research by Passioura (1983) considered that the more water consumption by a large root system could be offset by a decrease in the harvest index, which would moderately decrease the root/shoot ratio^38^. This result was further confirmed by Ma *et al*.^39^. Wojciechowski *et al*.^20^ and Waines and Ehdaie (2005)^40^ verified that the introduction of dwarf genes might affect the root weight during the revolution of modern wheat cultivars. Modern cultivars in Australia were confirmed to have smaller roots, reduced root redundancies, more roots in deep soil, and reduced root/shoot ratios, which resulted in more reasonable root configurations that were conducive to the improvement of agricultural production on dry land^41,42^. In addition, Allard *et al*. (2013) studied 16 wheat cultivars in France and reached the conclusion that the root/shoot ratio may not be an intrinsic attribute of wheat genotype^43^, as dry matter distribution in wheat was strongly influenced by tiller and water and fertilizer conditions and less affected by genotype. In this study, slight increases in root traits (root weight and root surface area) with decade were observed at both anthesis and maturity, but the results were not consistent for all root characteristics in all water treatments (Figs. 2–4). The stable increase in the root/shoot ratio with decade was confirmed at maturity, although the trend was not significant (P > 0.05). This result was very different from the results observed in previous studies^41^. According to a study by Passioura (1983)^38^, root weights of 500 kg ha^−1^ were sufficient for wheat to fully utilize the soil water at depths of 0–2 m. The root weights measured in this study are well beyond this value, and combined with the similar water consumption amounts for all cultivars with different root sizes, it could be speculated that there is severe root growth redundancy for dryland wheat in Shaanxi. The most likely cause might be the lack of studies on the influence of water deficit and fertilizer deficiency on the distribution of plant dry matter, as modern breeding work is often screened under sufficient water and fertilizer conditions^44^.

In this study, fluctuating changes in root surface area were observed; however, no consistent variations with decade were found (Figs. 5 and 6). The cultivars from the 2000s and 2010s showed larger root surface areas than the earlier cultivars at anthesis in the drought treatment in 2010–2011, especially at depths of 0–40 cm, while their root weights were not different from those of the other cultivars. A similar result was achieved in the drought treatment in 2011–2012, which might be due to the increase in root diameter^45^. Although the results did not stand out, they could reflect the ability of wheat to regulate more surface area and maintain a steady root weight in particular water conditions to benefit the extension of roots into the soil. The result of the increased WUE of modern cultivars (Fig. 9) was due to the increase in yield production^32^ rather than the change in root structure or the decrease in water consumption^46^. This result is not consistent with the results from dryland wheat in Australia from Siddique *et al*.^41^ and Aziz *et al*.^42^, which might be related to the different breeding requirements due to the different breeding strategies and growing conditions in different countries and regions.

Nakhforoosh *et al*.^13^ and Siddique *et al*.^41^ considered that more roots at the surface are beneficial for soil water consumption. However, larger root systems are associated with more radicles, shorter lives and greater dry matter losses after anthesis^43^. Evaporation from the soil surface might be too fast to allow absorption and utilization by plants, with roots unable to fully absorb soil water, leading to water consumption stability over decades (Fig. 8); thus, this condition is not conducive to improving the yield (Tables 3, 4) or WUE (Fig. 9). Furthermore, root system depth data suggested that cultivars with dwarf or semidwarf genes had more shallow root systems, confirming that deep roots are not essential for yield accumulation or WUE increase.

There is an urgent need to cultivate more drought-resistant cultivars in modern dryland wheat breeding^47^. The genotype differences in water sensitivity include not only the aboveground traits but also the root system traits^48^. Modern wheat varieties may have better abilities to adjust interbreed competition; thus, they can maintain a high yield^30^. In the experimental plot conditions in this study, the SSI of root traits (root weight and root surface area) at maturity of the landrace cultivar (1940s) was always the lowest or second-lowest among all cultivars (Fig. 7). As greater root systems were observed in the drought treatment than the irrigation treatment in this study, the landrace cultivar also showed smaller yield decreases in the drought treatment. The results illustrate that landrace cultivars might be more tolerant to water deficits. Modern cultivars showed more significant decreases in yields in the drought treatment, with higher SSI values than the older cultivars^32^, and further research is required on whether this difference is related to the smaller root increases in the drought treatment. For the cultivated cultivars, the SSI of roots decreased significantly from the 1950s to 2010s, meaning that the root systems of older cultivars are more sensitive to soil water conditions, while the modern cultivars showed decreased response intensities in the drought treatment, and the water sensitivity decreased. However, the SSI of modern cultivars was still higher than that of the landrace cultivar. Based on the above results, landrace cultivars might be more useful than other cultivars for dryland wheat breeding^49^.

## Conclusions

It can be concluded that the root weight and root surface area characteristics of dryland wheat cultivars in Shaanxi Province of China from the 1940s to 2010s were not consistent. The SSI of wheat roots in the drought treatment decreased from the 1950s to 2010s with breeding, but the SSI of the landrace cultivar from the 1940s was even lower than that of the modern cultivars. The modern cultivars after the 2000s possessed greater root surface areas than the older cultivars, which increased the expansion of roots in soil. However, the expansion was mainly at the surface from 0–40 cm, which resulted in similar water consumption values through the decades. The WUE of modern cultivars increased with the increase in the yield production without increasing the water consumption. The increase was limited as it was subjected to the root system traits. It is believed that paying more attention to root growth during the development of yield potential, optimizing the root/shoot relationship, increasing the root ratio in deep soil, and promoting the root vitality will be effective ways to increase the yields of dryland wheat in Shaanxi Province, China.
